# Personalized genome structure via single gamete sequencing

**DOI:** 10.1186/s13059-021-02327-w

**Published:** 2021-04-19

**Authors:** Ruqian Lyu, Vanessa Tsui, Davis J. McCarthy, Wayne Crismani

**Affiliations:** 1 Bioinformatics and Cellular Genomics, St. Vincent’s Institute of Medical Research, Melbourne, Australia; 2 Melbourne Integrative Genomics, Faculty of Science, The University of Melbourne, Melbourne, Australia; 3 DNA Repair and Recombination Laboratory, St. Vincent’s Institute of Medical Research, Melbourne, Australia; 4 The Faculty of Medicine, Dentistry and Health Science, The University of Melbourne, Melbourne, Australia

## Abstract

Genetic maps have been fundamental to building our understanding of disease genetics and evolutionary processes. The gametes of an individual contain all of the information required to perform a de novo chromosome-scale assembly of an individual’s genome, which historically has been performed with populations and pedigrees. Here, we discuss how single-cell gamete sequencing offers the potential to merge the advantages of short-read sequencing with the ability to build personalized genetic maps and open up an entirely new space in personalized genetics.

## Introduction

Reference genomes are valuable resources for biological research ranging from specific gene function through to studying evolution. After decades of investment, the high-quality human reference genome (GRCh38) has revolutionized clinical diagnostics. However, the human genome still contains gaps and only recently has a telomere-to-telomere assembly of a single human chromosome been within reach [1]. Nevertheless, a reference genome does not represent the vast genetic variation between any two individuals. The aggregation of genetic variation from multiple genomes is available through consortia (e.g., gnomAD), and graph genomes provide a useful way of integrating structural variation and reference genomes. The current laboratory methods used to assay genetic variation are often a combination of techniques such as bulk short- and long-read sequencing, optical mapping, and cytogenetics. A complementary tool for chromosome-scale assembly discussed here is the combination of accurate short-read sequencing applied to nuclear DNA from single gametes. This review is intended for a broad audience. Readers familiar with genetic linkage and genome assembly may wish to advance to the “Platforms for constructing iMaps—proof of principle and opportunities” section.

**Fig. 1:**
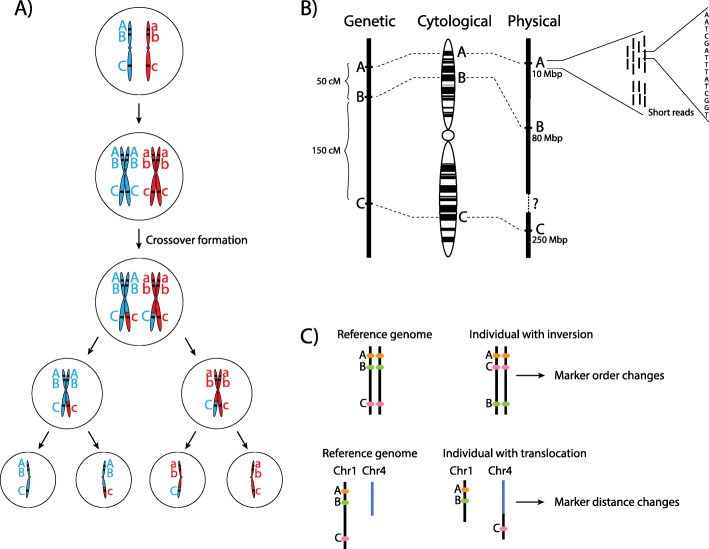
Meiosis and linkage. **a** Meiosis involves two rounds of cell divisions following DNA replication. In the first division, meiosis I, homologous chromosomes pair for crossover formation, creating a physical link (chiasmata), to exchange some genetic material and resulting in two haploid cells that have half the number of chromosomes as the original cell. Meiosis II occurs when the sister chromatids segregate to generate four genetically unique gametes (sperm or egg). **b** Comparison of genetic, cytological, and physical maps, all of which characterize genetic markers. A genetic map is based on the frequency of co-segregation of linked markers. A cytological map can be constructed by labeling certain DNA markers or particular staining methods. cM, centiMorgan; Mbp, megabase base pair of DNA. **c** An iMap with an inversion does not alter the DNA sequence but changes the linear ordering of markers. Translocation as a result of chromosome breakage and fusion affects crossover formation and changes the marker distance

High-throughput DNA sequencing has made genome assembly more accessible; however, fragmented DNA sequences still need to be assembled into highly contiguous chromosomal sequences. Genome assembly has historically required a dense genetic map to anchor and orient short DNA sequences onto larger chromosome-scale fragments. A genetic map is an ordering and spacing of loci (identified by markers) on a chromosome map (Fig. 1a) [2]. Genetic maps can be built for sexually reproducing species due to chromosome reshuffling in a process called meiosis. Meiosis serves two purposes: (1) the generation of haploid gametes, sperm, and eggs and (2) the genetic diversification of gametes, by chromosome segregation and meiotic crossovers (Fig. 1b). Meiotic crossovers (COs) are large reciprocal exchanges of genetic material between homologous chromosomes, which generate unique combinations of alleles [3]. Crossover frequencies between linked markers are used to calculate genetic distances, measured in centiMorgans, which enable marker ordering at a fine scale. Historically, genetic maps preceded physical chromosome maps, and physical sequence was anchored to its appropriate position on a genetic map. However, high-throughput sequencing revolutionized the analysis of genomes by generating orders of magnitude more data at a reasonable cost. In turn, marker density increased in line with sequencing capabilities rapidly changing how researchers assemble genomes. More recent advances in genome assembly include optical mapping, long-read technologies, strand-seq, and software capable of managing the assembly of large repetitive genomes [4–7]. A flow-on effect from the construction of marker-dense physical maps and genetic maps was an increased capacity to research the non-random distribution of crossovers throughout the human and mouse genomes [8–10].

## Genetic map variation between individuals: a motivation for personalized genetic maps

Genetic maps are population averages and, if personalized, would differ between any two individuals. These differences can be attributed to two reasons, both of which are challenging to assay: (1) there is structural variation between their chromosomes, and (2) the crossover distributions in their gametes differ due to genetic regulation.

### Crossover hotspot localization

The distribution of crossovers is not homogeneous across chromosomes, equally when considering a chromosome arm, or focusing down to a scale of hundreds of nucleotides. Research into the heterogeneity of crossover distributions led to the remarkable independent discoveries of PRDM9 as a major determinant of crossover hotspots in humans and mice. Different alleles of *PRDM9*, and the 13-mer to which it binds to initiate meiotic recombination, can explain a significant portion (~ 40%) of crossover hotspot localization in the genome when comparing across populations [11–17]. Further studies have continued to dissect the variation in crossover distributions down to the level of interpersonal heterogeneity [17–21].

### Heterochiasmy

Differences in crossover distributions also exist between males and females [12, 22, 23] and even male and female organs of hermaphrodite plants [24]. In addition, clusters of sex-specific recombination hotspots were found in different regions of the human genome [12, 21, 25, 26]. Male recombination occurs more frequently in exons of genes and telomeric regions, whereas in females, a higher proportion of crossovers occurs between genes and in promoter regions [12, 21]. Further, other factors play a role such as survivor bias. For example, children from older mothers have higher crossover rates compared to those from younger mothers, consistent with the idea that “extra” crossovers promote the retention of bivalents, and in turn protect against non-disjunction [27]. This is an area of research that would benefit from improved tools to study inter-individual crossover variation.

### Genomic structural variation

Structural variation (SV) is by definition a variation in the marker ordering of a genetic and physical map (Fig. 1c). SVs are genomic alterations that can range from chromosome-scale alterations down to smaller inversions, duplications, insertions, deletions, and translocations. Historically, chromosomal-scale SVs have been detected with cytogenetic approaches and can still only be visualized when multiple megabases in size. An orthogonal approach to detect SV, such as inversions, by measuring crossovers is possible by observing changes in marker linkage that can only be resolved through a reordering of markers. However, advances in high-throughput sequencing and computational methods are improving detection and breakpoint resolution [28].

The minimum size of an SV is arbitrary sometimes referring to alterations > 50 bp, but SVs are prevalent between any two individuals in the size ranges of hundreds of base pairs to multi-megabase scale [29–33]. One of the most comprehensive lists currently available of SVs identified in healthy humans has been obtained by combining several modern sequencing technologies to phase assembly of human genomes de novo, identifying > 27,000 SVs and > 150 inversions per genome [34].

Even with current short- and long-read sequencing technologies, SV detection is challenging above 100 kb [34, 35]. For example, inversions remain difficult to identify as inversion breakpoints typically reside in large repetitive regions and suppress crossovers, which are needed for their detection [34].

Different technologies generate datasets that offer different advantages in SV detection. Short-read sequencing has high single-nucleotide accuracy but lower sensitivity and higher false-positive rate in detecting SVs due to PCR amplification and low mappability to large repetitive sequences owing to shorter read lengths [34, 36]. Long-read technologies can partially overcome the shortcomings of investigating SVs using short-read sequencing thanks to their ability to span much longer sequences (up to 2 Mb) [37, 38]. The higher nucleotide error rate relative to short-read platforms has been a barrier for the adoption of long-read platforms for certain applications, but recent improvements in accuracy suggest a prominent role for long-read sequencing in calling SVs into the future [38–41].

Understanding the patterns and distribution of structural variation can facilitate appropriate interpretation of clinical diagnostic testing data for clinical applications and, more broadly, improve disease gene mapping. We speculate that haplotype-resolved individual or “personalized” genomes will become more clinically relevant and useful as the connection between SV and health advances to a point comparable to where gene function and disease is today.

### The iMap

**Fig. 2:**
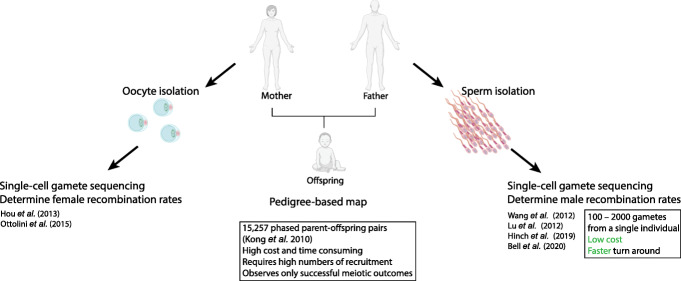
Pedigree-based maps and iMaps. Genetic maps can be constructed de novo using pedigree data. Personalized genetic maps can be inferred from gamete-derived sequence data

In eukaryotes, the number of crossovers is low per meiosis; therefore, large numbers of sequenced gametes are required to create a high-density genetic map. Single-cell sequencing enables the analysis of many thousands of genetically unique gametes from one person to generate an individual genetic map, which we term an iMap. The iMap overcomes the challenge of genotyping many human families with few offspring (Fig. 2). The potential benefits of being able to construct an iMap in any sexually reproducing species are significant. For human research, it will facilitate the detection of structural variation in challenging size ranges between average long-read lengths and cytogenetic resolution, e.g., 50 kb to 3 Mbp. The iMap could therefore be used as a complementary tool for genome completion and gap closing. The advantage of the iMap approach can be the capacity to assay tens of thousands of samples from one individual, and likely more as sequencing costs come down. Therefore, genomic regions that have extremely low crossover rates, e.g., centromeric regions, will still be challenging to assemble, but by assaying many gametes, researchers can increase the probability of finding the desired recombinants to assist with gap closing. Beyond human research, with common laboratory animals, the ethical implications are positive as a genetic map can be built from one animal rather than thousands. This approach will also reduce animal housing costs. Further, for exotic and endangered species, we speculate that these technologies will eventually bring benefits such as rapid de novo genetic map construction.

As chromosome numbers and ploidy increase, so too does the difficulty of finding linked markers for the construction of a genetic map. Gamete-derived iMaps offer a valuable component in the genome assembly toolkit. An important proof of concept with apricot used a droplet encapsulation technique to sequence haploid gametes [42]. Using 445 gametes, SNPs were phased using genetic linkage. Next, bulk long-read sequences were mapped to their matching haplotypes. A number of orthogonal approaches verified that an accurate de novo genome assembly had been generated. We believe that such an approach should assist consortia that sequence large, complex, and economically important genomes such as the 17-Gb allohexaploid bread wheat with 42 chromosomes [43–47]. Finally, the same apricot haploid short-read dataset also detected non-allelic meiotic crossovers at low frequencies, which opens up useful future fertility-related applications in individuals of all species. Short-read technologies alone cannot accurately detect and assemble the genome of species with high levels of duplications, and therefore, genome assembly requires an integrated approach.

## Platforms for constructing iMaps—proof of principle and opportunities

Single-cell technologies are advancing rapidly and can provide finer resolution measurement of cellular and molecular features of biological systems compared to bulk sequencing [48]. This technological advance has created opportunities for sequencing individual gametes at a large scale, which in turn allows the construction of iMaps. Human males, for example, can provide > 10^7^ spermatozoa [49] that can be collected non-invasively. Single-gamete sequencing studies that measure meiotic crossovers have emerged in the last decade [17–20, 50, 51]. We predict that this field will grow in the coming years with the increasing accessibility of the tools for single-cell isolation and sequencing that we review below.

**Fig. 3:**
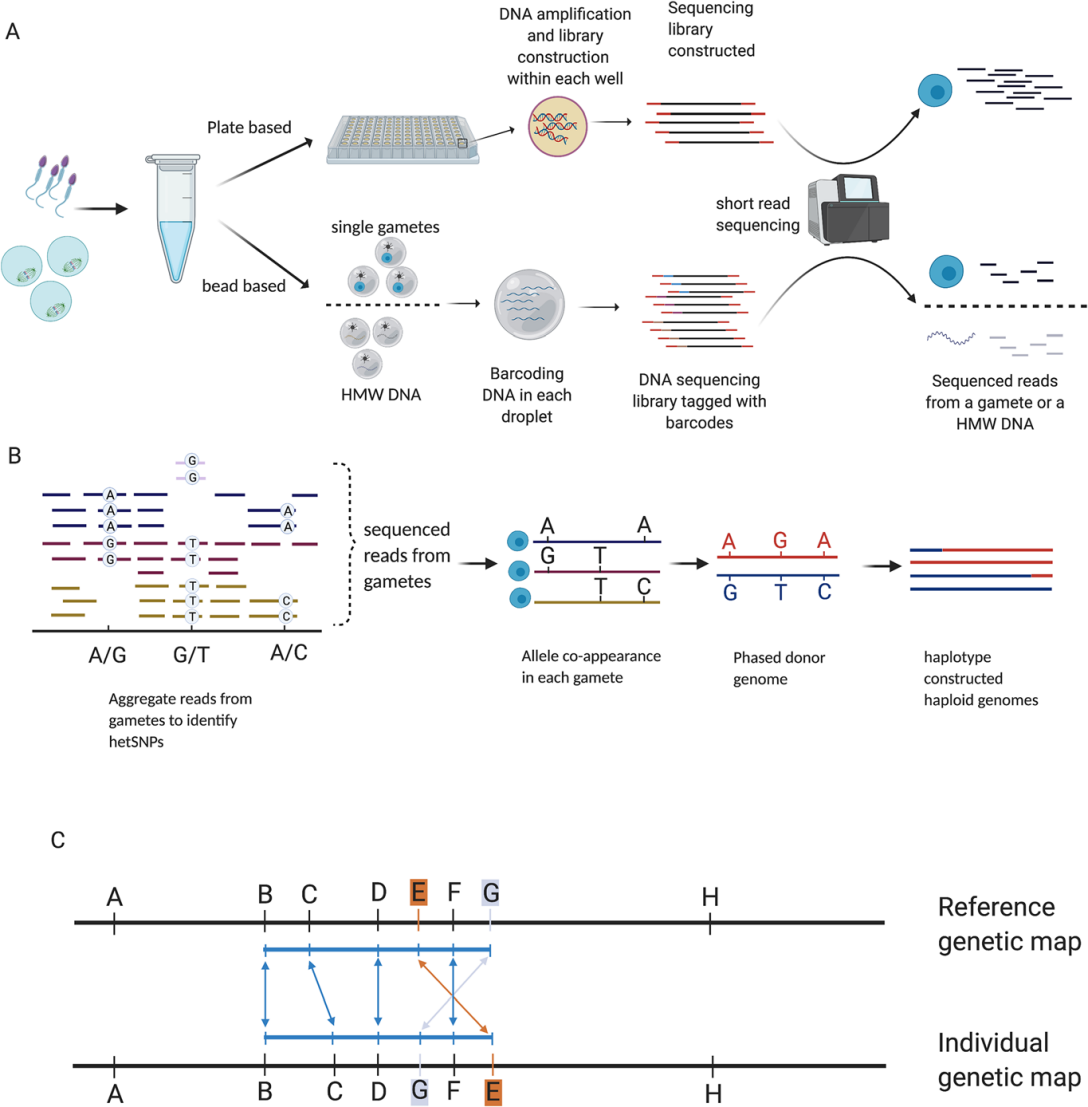
Representation of plate- and droplet-based methods for isolating and sequencing gametes and in silico map construction pipeline. **a** Schematic representation of plate- and bead-based approaches for profiling gametes. Gametes collected from a donor can be processed through plate-based methods; the single gamete is projected to individual compartments, and DNA amplification is carried out within each chamber for each gamete that can further be used for genotyping using SNP array or DNA sequencing. Bead-based methods either encapsulate single gametes or HMW DNA in a droplet with beads that contain a barcode. Pooled barcode-tagged reads are sequenced in parallel and provide gamete sources or HMW DNA sources. **b** General pipeline for crossover detection for individuals using gamete-based data. Reads from multiple gametes are aggregated for hetSNP identification. hetSNPs are phased based on SNP co-appearance in gametes. Genotypes of gametes and phased hetSNPs are used for constructing haplotypes of gametes that can be further used for crossover detection. **c** Illustration of marker ordering for an individual which shows different ordering and distancing from the reference genetic map

Sequencing single gametes is challenging due to the limitation of one copy of any given sequence per cell. Nevertheless, several methods exist for whole-genome amplification (WGA) of DNA from single cells that have been successfully applied for single-gamete sequencing and detection of crossovers. These methods include degenerate oligonucleotide primed polymerase chain reaction (DOP-PCR [52]), multiple displacement amplification (MDA [53]), multiple annealing and looping-based amplification cycles (MALBAC [54]), and, most recently, a method using RNA random priming [17]. Comparisons of amplification methods have been covered already [55, 56]. Here, we focus on potential applications of these methods for iMap construction that can be broadly grouped into plate-based or bead-based approaches and those that do or do not require amplification steps before library construction (Fig. 3). Further, we discuss the strengths and limitations of the different platforms (Table 1).

**Table 1:**
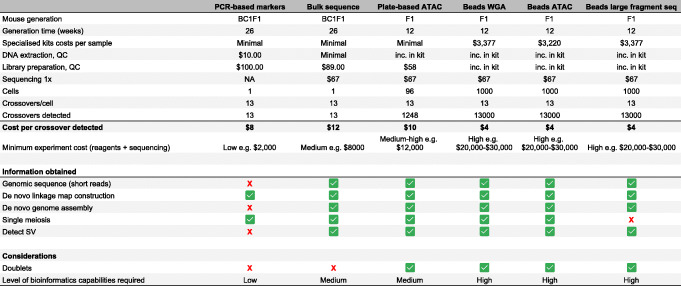
A comparison of the strengths and limitations of different methods for assaying crossovers. The assumptions used to build the table are the following: A male mouse would be used to obtain sperm. The mouse genome is approximately 2.5 Gbp, and generation times require 12 weeks. In bead-based single-cell experiments, 1000 gametes are captured and sequenced. 1× genome coverage is used in all sequence-based experiments. Representative costs have been used from experiments in our laboratories, or appropriate quotes, and are intended as a guide only. Costs are only for reagents and sequencing. Costs are not included for wet-lab and bioinformatics researchers, animal housing costs, and equipment.

### Plate-based gamete isolation methods

#### Multiple displacement amplification

Multiple displacement amplification (MDA) is an isothermal, strand-displacement amplification method that can provide more uniform amplification across the genome compared to PCR-based WGA methods [53] and was first applied to single-cell sequencing more than 15 years ago [57, 58]. MDA was integrated into a microfluidic device to capture single sperm cells and conduct whole-genome amplification of haploid genomes in parallel which, combined with multiplexing, can then be used for single-cell whole-genome sequencing [19]. By integrating MDA in the microfluidic device, the contamination and amplification-induced error rate were reduced compared to the original MDA method [19]. Crossover events and chromosome-level deletions could be detected in the 91 sperm amplification products produced, resulting in a personal recombination map that aligned well with averaged population map results [12, 59] while also revealing some individual-specific differences such as sub-telomeric and short-arm crossover frequencies.

#### Multiple annealing and looping-based amplification cycles

Multiple annealing and looping-based amplification cycles (MALBAC) for single-cell amplification further reduced amplification bias compared to MDA by introducing a quasi-linear pre-amplification step [54]. MALBAC has been used for haploid genome amplification and investigation of crossover distribution of an Asian donor through analyzing 99 sperm cells by first phasing the donor’s genome and then identifying crossovers in the sperm cells [18]. On average, 26 crossovers per sperm cell were identified, which is broadly consistent with population-scale average crossover estimates [22, 27] and cytological markers of crossovers per male meiosis [60, 61]. Crossover breakpoint locations could be identified with higher resolution using MALBAC rather than MDA. Aneupoid autosomes were found to have a significantly reduced crossover rate, but the same trend was not observed for aneuploid sex chromosomes [18]. A similar MALBAC approach to sequence individual sperm was used in a bull and compared to pedigree data from the same animal [62]. Good agreement in crossover distributions was observed speaking to the robustness of the iMap approach.

#### Whole-genome amplification via RNA random priming

RNA random priming has recently been proposed as a new method for linear whole-genome amplification in single sperm cells [17]. This method enables near-uniform genome coverage and further improves the resolution of detected crossovers relative to earlier methods [18, 19]. Whole genomes of 217 sperm cells from an F1 hybrid mouse (C57BL/6J X CAST/EiJ) with heterozygous *Prdm9* alleles (human/mouse) were sequenced, in combination with multiple molecular assays, to study factors that influence whether meiotic double-strand breaks (DSB) will be resolved as crossovers or non-crossovers. These data were used to assay crossover distributions of an individual in relation to chromosomal features, such as Prdm9 binding sites, distance from telomere, and local sequence GC content.

#### Key applications for plate-based platforms

Plate-based platforms offer lower throughput compared to bead-based platforms but allow a higher depth of coverage per cell and generally greater flexibility. Oocyte sequencing is one of the key applications for which plate-based platforms are appropriate given the low number of gametes that can be obtained from an individual. The oocyte pronucleus genomes were inferred by sequencing the first and second polar bodies, and the personalized female genetic map constructed was highly concordant with population-derived genetic maps (i.e., HapMap and deCODE) [50, 51]. For the first time, a personalized X chromosome recombination rate was estimated by sequencing, with the result (1.01–1.18 crossovers per meiosis) proving to be similar to crossover rate estimates for autosomes. Reduced crossover frequency in regions near transcription start sites was also observed, as well as reduced crossovers in aneuploid oocytes [18, 51], although the interpretation of these results must be tempered by the possibility of detection artifacts. The high genome coverage obtained with this plate-based approach enables detection of maternally derived aneuploidies and disease-associated single-nucleotide variants, which can aid in preimplantation genetic screening for healthy egg selection.

### Bead-based gamete isolation methods

#### Droplet encapsulation

A recently proposed method, *Sperm-seq*, can simultaneously sequence thousands of sperm genomes by encapsulating individual sperm cells in droplets using an integration of 10X Genomics and Drop-seq technologies [20]. With this approach, 31,228 sperm cells from 20 male human donors were collected and sequenced. Recombination rates in each sperm cell were estimated and then used to quantify genome-wide recombination frequencies for each donor individual. This study has by far the largest gamete sample size in profiling thousands of single sperm cells in parallel per donor, compared to previous studies that sequenced 100–200 sperm cells per donor. The large number of sperm cells analyzed from each single donor improves precision for the donor-specific crossover rate estimation.

#### Linked-reads sequencing

High-molecular weight (HMW) DNA molecules (~ 50 kb) can be fragmented, tagged, and computationally assembled by linked reads sequencing, which assists in haplotype phasing [63]. In the 10X Genomics system, HMW DNA molecules are partitioned and encapsulated in an emulsion droplet with barcodes attached to gel beads. Within each droplet, the HMW DNA is fragmented and barcoded (Fig. 3). Short-read sequencing can be used to sequence the barcoded DNA molecules as standard, and the barcode information can be exploited to assemble the long molecule [63–65]. Linked reads technology offers opportunities to measure crossover frequencies as demonstrated in mice, fish, and plants by pooling haploid genomes to detect recombinant molecules [64, 65]. The crossovers cannot be resolved at single gamete resolution but still could be used to measure crossover rates for the reconstruction of the linear order of genetic markers for an individual. Measuring crossover frequency in live progeny, e.g., F2 mice, or a droplet with a high-molecular weight molecule, e.g., from an F1 mouse, is fundamentally the same in that for a heterozygous region, some progeny (or high-molecular weight molecules) will be “parental” and some will be “recombinant.” The fraction which are recombinant can be used with a mapping function to calculate the centiMorgan distance between markers. The principles that Sturtevant used to order more than two markers on the same chromosome can be equally applied with high-molecular weight read recombination fractions. While the computational complexity is drastically larger when ordering hundreds of thousands of markers, the same principles apply.

### Challenges and opportunities

Bead-based methods all face the challenge of identifying and removing the effects of doublets, where two or more single cells (or DNA molecules, depending on the platform) are captured in the same physical droplet, and thus, the reads generated are tagged with the same barcode. In such cases, we no longer obtain single-cell information. A number of methods exist for doublet detection in droplet-based single-cell sequencing experiments [66–70]. In the construction of iMaps, unidentified doublets could cause false-positive crossover events to be called. However, unlike in most droplet-based single-cell applications, doublets in gamete sequencing do not necessarily mean that the corresponding barcodes (and cells that they represent) need to be discarded. Doublets can be identified for single-cell methods by metrics like heterozygosity (which is not expected when sequencing single haploid cells) and read number (more reads for a barcode are observed than would be expected for a single cell). For linked-read methods, the constructed DNA molecule size, coupled with the read number per constructed molecule, provides an indicator of doublet probability (for linked reads sequencing when two different HMW molecules, from adjacent genomic regions, are tagged with the same barcode and are incorrectly constructed as one continuous DNA molecule). While it is important to identify doublets in order to use the appropriate analysis tools, it is still possible to identify crossovers with doublet data.

While plate-based methods are less likely to generate doublets, bead-based methods are more readily applicable for large-scale studies owing to their suitability for batch processing of thousands to tens of thousands of cells in parallel. However, plate-based approaches offer more flexibility in adapting or optimizing experimental protocols as single cells are separated into wells. Thus, plate-based platforms offer greater opportunities for increasing sensitivity in molecule capture and, all else being equal, typically produce richer, higher-information data per cell.

The cost of whole-genome sequencing remains a challenge when profiling a large set of gametes. The potential of combining targeted sequencing, where only DNA regions of interest are amplified, with bead-based methods may enhance efficiency in detecting crossovers and reducing sequencing cost (Table 1). With continuing, rapid technological development in this field, deciding on the most efficient technology to detect crossovers in haploid cells and the sequencing depth required remain as open questions. With future improvements to sequencing depth and resolution, it may be possible to detect gene conversion events, which would be beneficial for crossover hotspot research. Specific experimental protocols and kits are in a constant state of flux as commercial solutions appear and disappear based on unpredictable market realities. Therefore, researchers may need to survey their options anew when undertaking studies such as those proposed here and may wish to consider the longevity and reliability of the access to the kits along with other technical features when choosing a platform for iMap data generation.

## Statistical inference and computational tools used in estimating crossover rates for building personalized genetic maps

Statistical inference methods are required to construct genetic maps from large datasets that identify crossovers. Here, we describe common statistical concepts that apply to the inference of crossover events from data collected from pedigree, population-level, and now individual-level single-gamete data.

In all natural populations, the observed linkage disequilibrium (LD) of alleles is shaped by generations of mutation and recombination, with specific LD patterns depending on the underlying sample characteristics. Building a genetic map from family- or pedigree-based cohorts takes advantage of co-segregation patterns of alleles across a known number of generations in families [12, 22, 71–73]. Coalescent-based statistical methods remain the method-of-choice for modeling the stochastic processes that make up population genetic histories and can incorporate estimation of population crossover rate [59, 74–76]. These methods provide either a population-averaged or a family-based estimation of crossover rates that cannot resolve a genetic map for each individual.

Here, we focus on gamete-based analysis pipelines that can be adopted for constructing personalized genomic structures from (single) gamete sequencing datasets, which will continue to accumulate with high-throughput sequencing and the advancement of single-cell sequencing methods (Platforms for constructing iMaps—proof of principle and opportunities).

### General pipeline

Construction of individual genetic maps from gametes starts with phasing the donor’s genome, that is defining the two haplotypes of the diploid donor genome. Crossover detection and genetic map construction based on crossover rate estimation can then follow.

#### Phasing

In some cases, the genome or genotype phase can be directly obtained from high-quality maternal and paternal reference genomes, such as when analyzing data from an F1 hybrid of two inbred strains that each have a genome assembly or known genotypes available [17, 64]. Otherwise, additional experiments such as deep sequencing of germline DNA samples can be conducted for phasing donor genomes.

In fact, the gametes’ haploid genomes contain sufficient information about the donor’s genome for haplotype reconstruction. Thus, especially when many gametes are collected, genotypes called from gametes can be aggregated to infer the phase of the donor [20, 51, 65, 77] (Fig. 3). To phase the donor genome, the first step is finding the hetSNPs (heterozygous SNP loci that differ between the maternal and paternal homologous chromosomes) in the donor’s genome by aligning DNA reads pooled from gametes to a reference genome and calling genotypes [51, 65]. The linkage of SNPs in each gamete is used for phasing the donor, that is the hetSNP genotypes that appear in the same gamete more often than expected by chance are inferred to lie on the same haplotype [18, 51]. The observation of genotypes in each gamete’s haploid genome is analogous to sequencing long fragments of (recombinant) chromosomes from the donor. Therefore, long fragment-based phasing tools can be applied to infer the phased genome for the individual [20, 65, 78, 79]. It is worth noting that iMap construction—like all genetic map construction—is most useful for genomic regions of heterozygosity.

#### Crossover detection

**Fig. 4:**
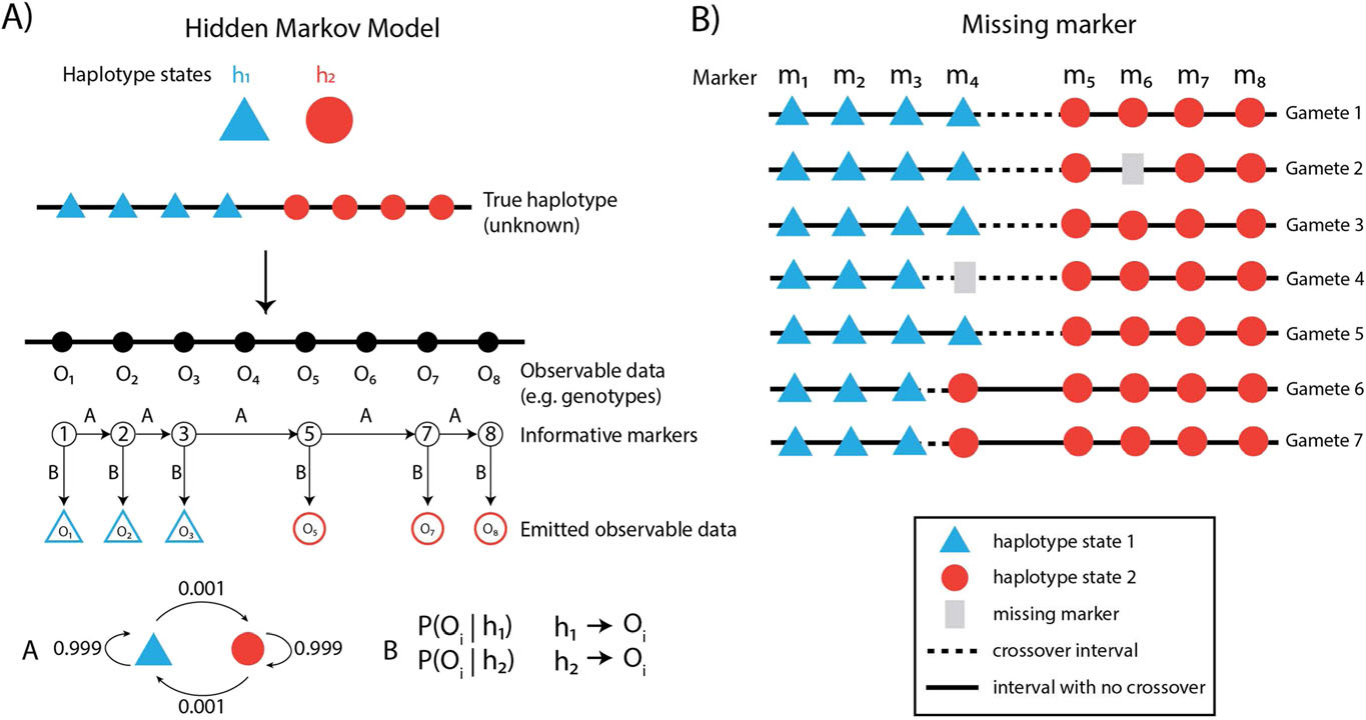
Statistical methods for crossover detection using a hidden Markov model. **a** The true haplotypes of the markers (h1, h2) are unknown, and the transitions between haplotype states are modeled by a hidden Markov model. The genotypes are observed from data and are controlled via an emission model **b**. Integrating information from the observed data, the transition model, and emission model, the most likely true haplotype sequence is inferred. **b** Example of a missing markers in Gamete 2 and Gamete 4. In Gamete 4 missing data for marker m4 creates ambiguity in crossover identification. Statistical inference methods can be used to probabilistically assign crossovers to the subinterval where information is missing

With genome phase of the donor known and called genotypes of hetSNPs in each gamete (or reconstructed DNA molecule), crossovers are inferred by detecting haplotype shifts along a chromosome from the gamete’s hetSNP genotypes or counting the number of recombinant DNA molecules (in the case of linked-reads sequencing). The following section focuses on how a haplotype shift can be detected—and therefore crossovers called—using a hidden Markov model (HMM; Fig. 4), a statistical model frequently applied in the analysis of genomic sequence data.

##### Hidden Markov Model

HMM approaches are commonly used in crossover detection due to the sequential nature of chromosomes [17, 80]. In the context of crossover inference, we want to know the true haplotypes of hetSNPs, which are modeled as hidden states, meaning that they are not directly observed but must be inferred. Instead of the true haplotypes, we observe the genotypes called at each hetSNP or, even more fundamentally, the sequencing reads overlapping hetSNPs that provide evidence for the allele inherited from one or the other parent (Fig. 4). Through modeling the transition between haplotype states and the probability of the observed data, the most probable state sequence can be derived, from which the crossovers are inferred. Crossover hotspots are located in various positions across the genome, which implies different crossover rates and hence transition probabilities between states. However, a homogeneous transition probability is often used in HMMs for crossover detection, because within one meiosis, the crossover detection should be driven by observed data instead of guided by prior hotspot localization knowledge. Aggregating crossover positions found in each meiosis leads to the identification of hotspots within an individual and across populations.

#### Genetic distance and map construction

The genetic distance (in units of centiMorgans) measures the likelihood of a crossover occurring between two markers and are computed based on the observed crossover rate. For short intervals between markers, where the recombination frequency is low, the genetic distances tend to be close to the raw crossover rate estimated. For larger crossover rates (observed recombination fraction > 0.1), mapping functions such as Haldane or Kosambi (see Glossary), constructed with different assumptions on crossover interference, are usually applied to adjust the larger crossover rates to additive genetic distance units. Even with the availability of dense marker sets in well-studied organisms, including humans, runs of homozygosity may lead to large enough marker intervals that mapping functions remain relevant for computing genetic distances from estimated crossover rates. Genetic distances measured from querying gametes produced from an individual lead to the construction of an iMap.

### Challenges and opportunities

#### Comparing genetic maps

To facilitate comparisons of genetic maps, genetic distances can be computed using a window-based approach, which bins chromosomes by genomic positions (using, e.g., 1 Mb interval bins) and calculates crossover frequency per bin [17, 18]. Correlation analysis among individual genetic maps or with a reference genetic map such as HapMap can be performed across the binned windows. However, the window size and the marker densities in different studies affect the comparisons and need to be managed carefully. Different window sizes may be tried to identify crossover events with higher or lower resolution. Larger window sizes will enable more stable identification of the haplotype but reduce the precision of inferred crossover locations. Higher marker density and deeper read coverage allow the use of smaller window sizes. Downsampling analysis can be helpful in making sure the marker densities are at the same scale across all samples [20]. Inter-individual differences in meiotic crossover landscapes can be revealed by crossover density plots that plot distributions of crossover locations along chromosomes for all individuals. Statistical tests for differences in distributions of the number of crossovers detected per gamete within individuals can also be applied. Such tests can be used to test the effects of specific factors that may influence variation in individual genetic maps. Bootstrapping and permutation testing approaches are useful where assumptions required for standard statistical estimators are not met, for example, when comparing total genetic map lengths or evidence for crossover interference between experimental groups. In addition, individualized crossover interference can be analyzed and compared within individuals [20].

### Missing markers

Marker-based crossover frequency estimation for each gamete faces the problem of missing markers in which some marker information may be missing from the underlying gamete (Fig. 4b), due to lack of reads or other factors. As in pedigree-based studies [12, 21], statistical inference can be used to improve the estimation of crossovers in marker intervals for single-gamete-based datasets.

When the observed crossover can only be assigned to a large marker interval (Fig. 4b, gamete 4, m3-m5) because information from markers (i.e., m4) within the interval is missing, a simple approach attributes the observed crossover to all the constituent intervals (m3-m4, m4-m5) within it evenly, or proportionally based on intervals’ physical sizes. A more sophisticated approach adopts the EM (expectation-maximization) algorithm which is a statistical algorithm for dealing with incomplete data with unobserved latent variables [81]. The EM algorithm uses information about crossover frequencies in sub-intervals from other samples for increased precision of crossover rate estimation when marker data is missing. It can be used to find the expected number of crossover events happening in each interval with some markers missing in certain meioses [12] (Fig. 4).

To infer the expected number of crossover events in each sub-interval and refine the estimation of crossover rates (for example, Fig. 4b, in gamete 4, m3-m4, m4-m5), the EM algorithm starts with an initial guess and updates the crossover membership probabilities to the sub-intervals iteratively. In each iteration, the crossover membership probabilities are updated based on crossover rates estimated from the previous step, and the crossover rates are updated with newly assigned crossover membership probabilities. When the estimates converge, that is show little change after some minimum number of iterations, the final estimates are obtained.

Another class of statistical inference methods uses simulation or sampling-based solutions that generate random samples from the desired distribution [21, 82]. For instance, a hierarchical model which assumed a Poisson distribution for crossover counts within each interval and a Gamma distribution for crossover frequencies for each interval has been used [21]. Estimation of refined crossover rates for marker intervals was achieved by implementing a Markov chain Monte Carlo-based approach (Gibbs sampler) that samples from the conditional distributions of relevant parameters.

EM- and MCMC-based methods both assume that individual meioses under analysis (assayed from gametes or whole organisms) share similar crossover positions along the list of markers. If the individual meioses are believed to have heterogeneous crossover landscapes, then these methods either should not be applied or, with caution, should be applied separately to sub-groups with similar expected landscapes.

## Conclusion and future perspectives

Genetic maps have provided a solid foundation for twenty-first century genome biology. The future of personalized genomics likely is a combination of long-read sequencing methods with specialized DNA library preparation methods (e.g., linked-reads, strand-seq, Hi-C, optical mapping), and short-read sequencing techniques that can provide accurate genotyping and reveal long-range chromosome-scale information. We believe that high-throughput sequencing of gametes offers a tool that can complement some of the limitations of other sequencing technologies. For example, iMaps may aid in patient screening for individuals who are at increased risk of having a pregnancy with an unbalanced genomic complement, and building personal genetic maps with higher physical resolution will help to accurately identify SVs. In addition, single-gamete sequencing could facilitate de novo genome assembly particularly for rare and endangered species.

## Supplementary Information

**Additional file 1.** Review history.

## References

[1] Miga KH, Koren S, Rhie A, Vollger MR, Gershman A, Bzikadze A, Brooks S, Howe E, Porubsky D, Logsdon GA, Schneider VA, Potapova T, Wood J, Chow W, Armstrong J, Fredrickson J, Pak E, Tigyi K, Kremitzki M, Markovic C, Maduro V, Dutra A, Bouffard GG, Chang AM, Hansen NF, Wilfert AB, Thibaud-Nissen F, Schmitt AD, Belton JM, Selvaraj S, Dennis MY, Soto DC, Sahasrabudhe R, Kaya G, Quick J, Loman NJ, Holmes N, Loose M, Surti U, Risques R, Graves Lindsay TA, Fulton R, Hall I, Paten B, Howe K, Timp W, Young A, Mullikin JC, Pevzner PA, Gerton JL, Sullivan BA, Eichler EE, Phillippy AM (2020). Telomere-to-telomere assembly of a complete human X chromosome. Nature..

[2] Sturtevant AH (1913). The linear arrangement of six sex-linked factors in Drosophila, as shown by their mode of association. J Exp Zool.

[3] Hunter N. Meiotic recombination: the essence of heredity. Cold Spring Harb Perspect Biol. 2015;7(12) Available from: 10.1101/cshperspect.a016618 10.1101/cshperspect.a016618 PMC4665078 26511629

[4] Rhie A, McCarthy SA, Fedrigo O, Damas J, Formenti G. Towards complete and error-free genome assemblies of all vertebrate species. bioRxiv. 2020; Available from: https://www.biorxiv.org/content/10.1101/2020.05.22.110833v1.full-text 10.1038/s41586-021-03451-0 PMC8081667 33911273

[5] Porubsky D, Ebert P, Audano PA, Vollger MR, Harvey WT, Marijon P, et al. Fully phased human genome assembly without parental data using single-cell strand sequencing and long reads. Nat Biotechnol. 2020; Available from: 10.1038/s41587-020-0719-5 10.1038/s41587-020-0719-5 PMC7954704 33288906

[6] Garg S, Fungtammasan A, Carroll A, Chou M, Schmitt A, Zhou X, et al. Chromosome-scale, haplotype-resolved assembly of human genomes. Nat Biotechnol. 2020; Available from: 10.1038/s41587-020-0711-0 10.1038/s41587-020-0711-0 PMC7954703 33288905

[7] Schrinner SD, Mari RS, Ebler J, Rautiainen M, Seillier L, Reimer JJ, Usadel B, Marschall T, Klau GW (2020). Haplotype threading: accurate polyploid phasing from long reads. Genome Biol.

[8] Paigen K, Szatkiewicz JP, Sawyer K, Leahy N, Parvanov ED, Ng SHS, Graber JH, Broman KW, Petkov PM (2008). The recombinational anatomy of a mouse chromosome. PLoS Genet.

[9] McVean GAT, Myers SR, Hunt S, Deloukas P, Bentley DR, Donnelly P (2004). The fine-scale structure of recombination rate variation in the human genome. Science..

[10] Myers S, Bottolo L, Freeman C, McVean G, Donnelly P (2005). A fine-scale map of recombination rates and hotspots across the human genome. Science..

[11] Berg IL, Neumann R, Sarbajna S, Odenthal-Hesse L, Butler NJ, Jeffreys AJ (2011). Variants of the protein PRDM9 differentially regulate a set of human meiotic recombination hotspots highly active in African populations. Proc Natl Acad Sci U S A.

[12] Kong A, Thorleifsson G, Gudbjartsson DF, Masson G, Sigurdsson A, Jonasdottir A, Walters GB, Jonasdottir A, Gylfason A, Kristinsson KT, Gudjonsson SA, Frigge ML, Helgason A, Thorsteinsdottir U, Stefansson K (2010). Fine-scale recombination rate differences between sexes, populations and individuals. Nature..

[13] Berg IL, Neumann R, Lam K-WG, Sarbajna S, Odenthal-Hesse L, May CA, Jeffreys AJ (2010). PRDM9 variation strongly influences recombination hot-spot activity and meiotic instability in humans. Nat Genet.

[14] Parvanov ED, Petkov PM, Paigen K (2010). Prdm9 controls activation of mammalian recombination hotspots. Science..

[15] Myers S, Bowden R, Tumian A, Bontrop RE, Freeman C, MacFie TS (2010). Drive against hotspot motifs in primates implicates the PRDM9 gene in meiotic recombination. Science..

[16] Baudat F, Buard J, Grey C, Fledel-Alon A, Ober C, Przeworski M, Coop G, de Massy B (2010). PRDM9 is a major determinant of meiotic recombination hotspots in humans and mice. Science..

[17] Hinch AG, Zhang G, Becker PW, Moralli D, Hinch R, Davies B, et al. Factors influencing meiotic recombination revealed by whole-genome sequencing of single sperm. Science. 2019;363(6433) Available from: 10.1126/science.aau8861 10.1126/science.aau8861 PMC6445350 30898902

[18] Lu S, Zong C, Fan W, Yang M, Li J, Chapman AR, Zhu P, Hu X, Xu L, Yan L, Bai F, Qiao J, Tang F, Li R, Xie XS (2012). Probing meiotic recombination and aneuploidy of single sperm cells by whole-genome sequencing. Science..

[19] Wang J, Fan HC, Behr B, Quake SR (2012). Genome-wide single-cell analysis of recombination activity and de novo mutation rates in human sperm. Cell..

[20] Bell AD, Mello CJ, Nemesh J, Brumbaugh SA, Wysoker A, McCarroll SA (2020). Insights into variation in meiosis from 31,228 human sperm genomes. Nature..

[21] Bhérer C, Campbell CL, Auton A (2017). Refined genetic maps reveal sexual dimorphism in human meiotic recombination at multiple scales. Nat Commun.

[22] Kong A, Gudbjartsson DF, Sainz J, Jonsdottir GM, Gudjonsson SA, Richardsson B, Sigurdardottir S, Barnard J, Hallbeck B, Masson G, Shlien A, Palsson ST, Frigge ML, Thorgeirsson TE, Gulcher JR, Stefansson K (2002). A high-resolution recombination map of the human genome. Nat Genet.

[23] Broman KW, Murray JC, Sheffield VC, White RL, Weber JL (1998). Comprehensive human genetic maps: individual and sex-specific variation in recombination. Am J Hum Genet.

[24] Giraut L, Falque M, Drouaud J, Pereira L, Martin OC, Mézard C (2011). Genome-wide crossover distribution in Arabidopsis thaliana meiosis reveals sex-specific patterns along chromosomes. PLoS Genet.

[25] de Boer E, Jasin M, Keeney S (2015). Local and sex-specific biases in crossover vs. noncrossover outcomes at meiotic recombination hot spots in mice. Genes Dev.

[26] Coop G, Wen X, Ober C, Pritchard JK, Przeworski M (2008). High-resolution mapping of crossovers reveals extensive variation in fine-scale recombination patterns among humans. Science..

[27] Campbell CL, Furlotte NA, Eriksson N, Hinds D, Auton A (2015). Escape from crossover interference increases with maternal age. Nat Commun.

[28] Mahmoud M, Gobet N, Cruz-Dávalos DI, Mounier N, Dessimoz C, Sedlazeck FJ (2019). Structural variant calling: the long and the short of it. Genome Biol.

[29] Kidd JM, Cooper GM, Donahue WF, Hayden HS, Sampas N, Graves T, Hansen N, Teague B, Alkan C, Antonacci F, Haugen E, Zerr T, Yamada NA, Tsang P, Newman TL, Tüzün E, Cheng Z, Ebling HM, Tusneem N, David R, Gillett W, Phelps KA, Weaver M, Saranga D, Brand A, Tao W, Gustafson E, McKernan K, Chen L, Malig M, Smith JD, Korn JM, McCarroll SA, Altshuler DA, Peiffer DA, Dorschner M, Stamatoyannopoulos J, Schwartz D, Nickerson DA, Mullikin JC, Wilson RK, Bruhn L, Olson MV, Kaul R, Smith DR, Eichler EE (2008). Mapping and sequencing of structural variation from eight human genomes. Nature..

[30] de Smith AJ, Tsalenko A, Sampas N, Scheffer A, Yamada NA, Tsang P, Ben-Dor A, Yakhini Z, Ellis RJ, Bruhn L, Laderman S, Froguel P, Blakemore AIF (2007). Array CGH analysis of copy number variation identifies 1284 new genes variant in healthy white males: implications for association studies of complex diseases. Hum Mol Genet.

[31] Stefansson H, Helgason A, Thorleifsson G, Steinthorsdottir V, Masson G, Barnard J, Baker A, Jonasdottir A, Ingason A, Gudnadottir VG, Desnica N, Hicks A, Gylfason A, Gudbjartsson DF, Jonsdottir GM, Sainz J, Agnarsson K, Birgisdottir B, Ghosh S, Olafsdottir A, Cazier JB, Kristjansson K, Frigge ML, Thorgeirsson TE, Gulcher JR, Kong A, Stefansson K (2005). A common inversion under selection in Europeans. Nat Genet.

[32] Telenti A, Pierce LCT, Biggs WH, di Iulio J, Wong EHM, Fabani MM, Kirkness EF, Moustafa A, Shah N, Xie C, Brewerton SC, Bulsara N, Garner C, Metzker G, Sandoval E, Perkins BA, Och FJ, Turpaz Y, Venter JC (2016). Deep sequencing of 10,000 human genomes. Proc Natl Acad Sci U S A.

[33] Audano PA, Sulovari A, Graves-Lindsay TA, Cantsilieris S, Sorensen M, Welch AE (2019). Characterizing the major structural variant alleles of the human genome. Cell.

[34] Chaisson MJP, Sanders AD, Zhao X, Malhotra A, Porubsky D, Rausch T, Gardner EJ, Rodriguez OL, Guo L, Collins RL, Fan X, Wen J, Handsaker RE, Fairley S, Kronenberg ZN, Kong X, Hormozdiari F, Lee D, Wenger AM, Hastie AR, Antaki D, Anantharaman T, Audano PA, Brand H, Cantsilieris S, Cao H, Cerveira E, Chen C, Chen X, Chin CS, Chong Z, Chuang NT, Lambert CC, Church DM, Clarke L, Farrell A, Flores J, Galeev T, Gorkin DU, Gujral M, Guryev V, Heaton WH, Korlach J, Kumar S, Kwon JY, Lam ET, Lee JE, Lee J, Lee WP, Lee SP, Li S, Marks P, Viaud-Martinez K, Meiers S, Munson KM, Navarro FCP, Nelson BJ, Nodzak C, Noor A, Kyriazopoulou-Panagiotopoulou S, Pang AWC, Qiu Y, Rosanio G, Ryan M, Stütz A, Spierings DCJ, Ward A, Welch AME, Xiao M, Xu W, Zhang C, Zhu Q, Zheng-Bradley X, Lowy E, Yakneen S, McCarroll S, Jun G, Ding L, Koh CL, Ren B, Flicek P, Chen K, Gerstein MB, Kwok PY, Lansdorp PM, Marth GT, Sebat J, Shi X, Bashir A, Ye K, Devine SE, Talkowski ME, Mills RE, Marschall T, Korbel JO, Eichler EE, Lee C (2019). Multi-platform discovery of haplotype-resolved structural variation in human genomes. Nat Commun.

[35] Huddleston J, Chaisson MJP, Steinberg KM, Warren W, Hoekzema K, Gordon D, Graves-Lindsay TA, Munson KM, Kronenberg ZN, Vives L, Peluso P, Boitano M, Chin CS, Korlach J, Wilson RK, Eichler EE (2017). Discovery and genotyping of structural variation from long-read haploid genome sequence data. Genome Res.

[36] De Coster W, Van Broeckhoven C (2019). Newest methods for detecting structural variations. Trends Biotechnol.

[37] Huddleston J, Eichler EE (2016). An incomplete understanding of human genetic variation. Genetics..

[38] Rang FJ, Kloosterman WP, de Ridder J (2018). From squiggle to basepair: computational approaches for improving nanopore sequencing read accuracy. Genome Biol.

[39] Jain M, Koren S, Miga KH, Quick J, Rand AC, Sasani TA, Tyson JR, Beggs AD, Dilthey AT, Fiddes IT, Malla S, Marriott H, Nieto T, O’Grady J, Olsen HE, Pedersen BS, Rhie A, Richardson H, Quinlan AR, Snutch TP, Tee L, Paten B, Phillippy AM, Simpson JT, Loman NJ, Loose M (2018). Nanopore sequencing and assembly of a human genome with ultra-long reads. Nat Biotechnol.

[40] Wenger AM, Peluso P, Rowell WJ, Chang P-C, Hall RJ, Concepcion GT, Ebler J, Fungtammasan A, Kolesnikov A, Olson ND, Töpfer A, Alonge M, Mahmoud M, Qian Y, Chin CS, Phillippy AM, Schatz MC, Myers G, DePristo MA, Ruan J, Marschall T, Sedlazeck FJ, Zook JM, Li H, Koren S, Carroll A, Rank DR, Hunkapiller MW (2019). Accurate circular consensus long-read sequencing improves variant detection and assembly of a human genome. Nat Biotechnol.

[41] Amarasinghe SL, Su S, Dong X, Zappia L, Ritchie ME, Gouil Q (2020). Opportunities and challenges in long-read sequencing data analysis. Genome Biol.

[42] Campoy JA, Sun H, Goel M, Jiao W-B, Folz-Donahue K, Wang N, Rubio M, Liu C, Kukat C, Ruiz D, Huettel B, Schneeberger K (2020). Gamete binning: chromosome-level and haplotype-resolved genome assembly enabled by high-throughput single-cell sequencing of gamete genomes. Genome Biol.

[43] Paux E, Sourdille P, Salse J, Saintenac C, Choulet F, Leroy P, Korol A, Michalak M, Kianian S, Spielmeyer W, Lagudah E, Somers D, Kilian A, Alaux M, Vautrin S, Berges H, Eversole K, Appels R, Safar J, Simkova H, Dolezel J, Bernard M, Feuillet C (2008). A physical map of the 1-gigabase bread wheat chromosome 3B. Science..

[44] Ling H-Q, Zhao S, Liu D, Wang J, Sun H, Zhang C, Fan H, Li D, Dong L, Tao Y, Gao C, Wu H, Li Y, Cui Y, Guo X, Zheng S, Wang B, Yu K, Liang Q, Yang W, Lou X, Chen J, Feng M, Jian J, Zhang X, Luo G, Jiang Y, Liu J, Wang Z, Sha Y, Zhang B, Wu H, Tang D, Shen Q, Xue P, Zou S, Wang X, Liu X, Wang F, Yang Y, An X, Dong Z, Zhang K, Zhang X, Luo MC, Dvorak J, Tong Y, Wang J, Yang H, Li Z, Wang D, Zhang A, Wang J (2013). Draft genome of the wheat A-genome progenitor Triticum urartu. Nature..

[45] Ling H-Q, Ma B, Shi X, Liu H, Dong L, Sun H, Cao Y, Gao Q, Zheng S, Li Y, Yu Y, du H, Qi M, Li Y, Lu H, Yu H, Cui Y, Wang N, Chen C, Wu H, Zhao Y, Zhang J, Li Y, Zhou W, Zhang B, Hu W, van Eijk MJT, Tang J, Witsenboer HMA, Zhao S, Li Z, Zhang A, Wang D, Liang C (2018). Genome sequence of the progenitor of wheat A subgenome Triticum urartu. Nature..

[46] International Wheat Genome Sequencing Consortium (IWGSC), IWGSC RefSeq principal investigators, Appels R, Eversole K, Feuillet C, Keller B, et al. Shifting the limits in wheat research and breeding using a fully annotated reference genome. Science. 2018;361(6403) Available from: 10.1126/science.aar7191 10.1126/science.aar7191 30115783

[47] International Wheat Genome Sequencing Consortium (IWGSC) (2014). A chromosome-based draft sequence of the hexaploid bread wheat (*Triticum aestivum*) genome. Science.

[48] Stuart T, Satija R (2019). Integrative single-cell analysis. Nat Rev Genet..

[49] Cooper TG, Noonan E, von Eckardstein S, Auger J, Baker HWG, Behre HM, Haugen TB, Kruger T, Wang C, Mbizvo MT, Vogelsong KM (2010). World Health Organization reference values for human semen characteristics. Hum Reprod Update.

[50] Ottolini CS, Newnham L, Capalbo A, Natesan SA, Joshi HA, Cimadomo D (2015). Genome-wide maps of recombination and chromosome segregation in human oocytes and embryos show selection for maternal recombination rates. Nat Genet.

[51] Hou Y, Fan W, Yan L, Li R, Lian Y, Huang J, Li J, Xu L, Tang F, Xie XS, Qiao J (2013). Genome analyses of single human oocytes. Cell..

[52] Telenius H, Carter NP, Bebb CE, Nordenskjöld M, Ponder BA, Tunnacliffe A (1992). Degenerate oligonucleotide-primed PCR: general amplification of target DNA by a single degenerate primer. Genomics..

[53] Dean FB, Hosono S, Fang L, Wu X, Faruqi AF, Bray-Ward P, Sun Z, Zong Q, du Y, du J, Driscoll M, Song W, Kingsmore SF, Egholm M, Lasken RS (2002). Comprehensive human genome amplification using multiple displacement amplification. Proc Natl Acad Sci U S A.

[54] Zong C, Lu S, Chapman AR, Xie XS (2012). Genome-wide detection of single-nucleotide and copy-number variations of a single human cell. Science..

[55] de Bourcy CFA, De Vlaminck I, Kanbar JN, Wang J, Gawad C, Quake SR (2014). A quantitative comparison of single-cell whole genome amplification methods. PLoS One.

[56] Hou Y, Wu K, Shi X, Li F, Song L, Wu H, Dean M, Li G, Tsang S, Jiang R, Zhang X, Li B, Liu G, Bedekar N, Lu N, Xie G, Liang H, Chang L, Wang T, Chen J, Li Y, Zhang X, Yang H, Xu X, Wang L, Wang J (2015). Comparison of variations detection between whole-genome amplification methods used in single-cell resequencing. Gigascience..

[57] Handyside AH, Robinson MD, Simpson RJ, Omar MB, Shaw M-A, Grudzinskas JG, Rutherford A (2004). Isothermal whole genome amplification from single and small numbers of cells: a new era for preimplantation genetic diagnosis of inherited disease. Mol Hum Reprod.

[58] Hellani A, Coskun S, Benkhalifa M, Tbakhi A, Sakati N, Al-Odaib A (2004). Multiple displacement amplification on single cell and possible PGD applications. Mol Hum Reprod.

[59] International HapMap Consortium (2005). A haplotype map of the human genome. Nature..

[60] Barlow AL, Hultén MA (1998). Crossing over analysis at pachytene in man. Eur J Hum Genet.

[61] Gruhn JR, Rubio C, Broman KW, Hunt PA, Hassold T (2013). Cytological studies of human meiosis: sex-specific differences in recombination originate at, or prior to, establishment of double-strand breaks. PLoS One.

[62] Zhou Y, Shen B, Jiang J, Padhi A, Park K-E, Oswalt A, Sattler CG, Telugu BP, Chen H, Cole JB, Liu GE, Ma L (2018). Construction of PRDM9 allele-specific recombination maps in cattle using large-scale pedigree analysis and genome-wide single sperm genomics. DNA Res.

[63] Marks P, Garcia S, Barrio AM, Belhocine K, Bernate J, Bharadwaj R, Bjornson K, Catalanotti C, Delaney J, Fehr A, Fiddes IT, Galvin B, Heaton H, Herschleb J, Hindson C, Holt E, Jabara CB, Jett S, Keivanfar N, Kyriazopoulou-Panagiotopoulou S, Lek M, Lin B, Lowe A, Mahamdallie S, Maheshwari S, Makarewicz T, Marshall J, Meschi F, O’Keefe CJ, Ordonez H, Patel P, Price A, Royall A, Ruark E, Seal S, Schnall-Levin M, Shah P, Stafford D, Williams S, Wu I, Xu AW, Rahman N, MacArthur D, Church DM (2019). Resolving the full spectrum of human genome variation using linked-reads. Genome Res.

[64] Sun H, Rowan BA, Flood PJ, Brandt R, Fuss J, Hancock AM, Michelmore RW, Huettel B, Schneeberger K (2019). Linked-read sequencing of gametes allows efficient genome-wide analysis of meiotic recombination. Nat Commun.

[65] Dréau A, Venu V, Avdievich E, Gaspar L, Jones FC (2019). Genome-wide recombination map construction from single individuals using linked-read sequencing. Nat Commun.

[66] Wolock SL, Lopez R, Klein AM (2019). Scrublet: computational identification of cell doublets in single-cell transcriptomic data. Cell Syst.

[67] Bernstein NJ, Fong NL, Lam I, Roy MA, Hendrickson DG, Kelley DR (2020). Solo: doublet identification in single-cell RNA-Seq via semi-supervised deep learning. Cell Syst.

[68] DePasquale EAK, Schnell DJ, Van Camp P-J, Valiente-Alandí Í, Blaxall BC, Grimes HL (2019). DoubletDecon: deconvoluting doublets from single-cell RNA-sequencing data. Cell Rep.

[69] McGinnis CS, Murrow LM, Gartner ZJ (2019). DoubletFinder: doublet detection in single-cell RNA sequencing data using artificial nearest neighbors. Cell Syst.

[70] Bais AS, Kostka D. scds: computational annotation of doublets in single-cell RNA sequencing data. Bioinformatics. 2019; Available from: 10.1093/bioinformatics/btz698 10.1093/bioinformatics/btz698 PMC7703774 31501871

[71] Lander ES, Green P (1987). Construction of multilocus genetic linkage maps in humans. Proc Natl Acad Sci U S A.

[72] Gudbjartsson DF, Jonasson K, Frigge ML, Kong A (2000). Allegro, a new computer program for multipoint linkage analysis. Nat Genet.

[73] Abecasis GR, Wigginton JE (2005). Handling marker-marker linkage disequilibrium: pedigree analysis with clustered markers. Am J Hum Genet.

[74] Fearnhead P, Donnelly P (2001). Estimating recombination rates from population genetic data. Genetics..

[75] Fearnhead P, Harding RM, Schneider JA, Myers S, Donnelly P (2004). Application of coalescent methods to reveal fine-scale rate variation and recombination hotspots. Genetics..

[76] Stumpf MPH, McVean GAT (2003). Estimating recombination rates from population-genetic data. Nat Rev Genet.

[77] Kirkness EF, Grindberg RV, Yee-Greenbaum J, Marshall CR, Scherer SW, Lasken RS, Venter JC (2013). Sequencing of isolated sperm cells for direct haplotyping of a human genome. Genome Res.

[78] Bansal V, Bafna V (2008). HapCUT: an efficient and accurate algorithm for the haplotype assembly problem. Bioinformatics..

[79] Xie M, Wang J, Jiang T (2012). A fast and accurate algorithm for single individual haplotyping. BMC Syst Biol.

[80] Rowan BA, Patel V, Weigel D, Schneeberger K (2015). Rapid and inexpensive whole-genome genotyping-by-sequencing for crossover localization and fine-scale genetic mapping. G3.

[81] Dempster AP, Laird NM, Rubin DB (1977). Maximum likelihood from incomplete data via the EM algorithm. J R Stat Soc Series B Stat Methodol.

[82] Hinch AG, Tandon A, Patterson N, Song Y, Rohland N, Palmer CD, Chen GK, Wang K, Buxbaum SG, Akylbekova EL, Aldrich MC, Ambrosone CB, Amos C, Bandera EV, Berndt SI, Bernstein L, Blot WJ, Bock CH, Boerwinkle E, Cai Q, Caporaso N, Casey G, Adrienne Cupples L, Deming SL, Ryan Diver W, Divers J, Fornage M, Gillanders EM, Glessner J, Harris CC, Hu JJ, Ingles SA, Isaacs W, John EM, Linda Kao WH, Keating B, Kittles RA, Kolonel LN, Larkin E, le Marchand L, McNeill LH, Millikan RC, Murphy, Musani S, Neslund-Dudas C, Nyante S, Papanicolaou GJ, Press MF, Psaty BM, Reiner AP, Rich SS, Rodriguez-Gil JL, Rotter JI, Rybicki BA, Schwartz AG, Signorello LB, Spitz M, Strom SS, Thun MJ, Tucker MA, Wang Z, Wiencke JK, Witte JS, Wrensch M, Wu X, Yamamura Y, Zanetti KA, Zheng W, Ziegler RG, Zhu X, Redline S, Hirschhorn JN, Henderson BE, Taylor Jr HA, Price AL, Hakonarson H, Chanock SJ, Haiman CA, Wilson JG, Reich D, Myers SR (2011). The landscape of recombination in African Americans. Nature..

