# Supplementary material for: Personalized genome structure via single gamete sequencing
Source: Genome Biol. 2021 Apr 19;22:112. doi: 10.1186/s13059-021-02327-w (PMC8054432; doi:10.1186/s13059-021-02327-w)
Supplement: Supplementary file 1 — Additional file 1. Review history. [file 13059_2021_2327_MOESM1_ESM.docx]

Review History

**First round of review**

**Reviewer 1**

**Comments to author:**

This is an interesting review article that highlights the potential for gamete sequencing to generate 'iMaps' - or individual genetic maps, that would be valuable in revealing SVs, due to their effect on meiotic crossovers. The emphasis is on single gamete sequencing in bulk to generate large amounts of crossover data to allow generation of classical genetic maps (heterozygosity permitting). Overall the review does an excellent job of highlighting the state of the art and the benefits of these approaches as a complement to physical genome assembly methods.

I only have a few minor suggestions for revision.

1. Please cite Rowan et al G3 Rapid and Inexpensive Whole-Genome Genotyping-by-Sequencing for Crossover Localization and Fine-Scale Genetic Mapping, which reports a useful HMM for crossover detection from short read data - this has been quite widely applied in the plant field.

2. It is worth noting that the number of crossovers per chromosome per meiosis is usually low (1-2) meaning that large numbers of cells are required to build up a deep genetic map.

3. Please make it clear that this won't work very well if an individual is quite homozygous. Just out of curiosity how heterozygous is the 'typical' human?

4. The main focus is on crossovers - please also discuss gene conversions and the relative difficulty of detecting them due to short tract lengths. My feeling is that very low coverage data, which single cell data often is, is unsuitable for detection of conversions. I realise the gene conversions are not as relevant for SV detection, but they are also an interesting repair outcome from meiosis that would be interesting to study with single gamete approaches.

5. Are the authors aware of any examples of SV heterozygosity causing effects on crossovers in human pedigree or other classical genetic maps?

**Reviewer 2**

**Comments to author:**

The manuscript, through reviewing existing work, decries the idea of using single gamete sequencing to facilitate genetic map construction, which can subsequently help achieve haplotype-resolved assemblies for individuals, providing the basis for finding potential links between genetic variations and health. The authors explain technologies/methods and questions relevant to genetic map construction. Below lists my major and minor comments.

Major:

1. To avoid any misunderstanding, all concepts described in the manuscript need to be accurately defined. For example, what does genome structure (given in title), genetic structure (line 307) and haplotype structure (line 482) stand for?

2. The relationship between genome structure, genetic map and genome assembly need to be clearly and sufficiently explained.

3. Independent of a genetic map, highly continuous genome assembly can be achieved using other technologies, such as chromosome conformation capture (https://www.nature.com/articles/s41587-020-0711-0) or strand-seq (https://www.nature.com/articles/s41587-020-0719-5). Indeed, conversely, such assemblies can be viewed as genetic maps with a higher resolution than those built up with limited markers. Therefore, it would be necessary for the authors to discuss more on the advantages (and/or disadvantages) of using a genetic map to assist genome assembly, compared with other methods, to make the statements from line 37-54 reasonable.

4. As stated by the authors, there are variations in individual genetic maps, regarding crossover distributions (in gametes) and structural variations. It would be interesting to illustrate/propose the ideas of how such variations can be recognized and compared in details (although already stated briefly in line 372-378). For existing methods, comprehensively categorizing them (probably with a table) with advantages and disadvantages would be more helpful for readers.

5. It is doubtful that DNA molecule size and read density can be used to filter out "doublets" in single-cell sequencing (line 259-261). For example, how to reconstruct the molecules? It is not clear. However, if these would be true, could the authors explain ideas in details? And, besides "doublets", would "halflets" where DNA from the same cell are ligated with two different barcodes challenge the related analysis?

6. It would be interesting to discuss how feasible it is to build iMap in species with complex genome features, such as high levels of repeats.

Minor:

Line 103-105: please explain why crossovers are needed for characterizing inversions.

Line 124-149: "single-cell sequencing" is "single-cell whole-genome DNA sequencing", correct? Would there be other possibilities (related to lines 279-281), and would there be additional challenges?

Line 128-131: it needs to be elaborated more on how to achieve genome completion and gap closing using iMap. What would happen in highly repetitive regions like centromeres where there is nearly no recombination?

Line 254-256: Could the authors explain the general idea on how to reconstruct "linear order of genetic markers for an individual" with COs detected in pooled gametes?

Line 266-268: For polyploids, would there be ways to filter out doublets?

Line 377-378: What could be the measures on tuning window size and marker density? It's not informative by just saying "need to be managed carefully".

**Authors Response**

**Point-by-point responses to the reviewers’ comments:**

Reviewer #1: This is an interesting review article that highlights the potential for gamete sequencing to generate 'iMaps' - or individual genetic maps, that would be valuable in revealing SVs, due to their effect on meiotic crossovers. The emphasis is on single gamete sequencing in bulk to generate large amounts of crossover data to allow generation of classical genetic maps (heterozygosity permitting). Overall the review does an excellent job of highlighting the state of the art and the benefits of these approaches as a complement to physical genome assembly methods.

I only have a few minor suggestions for revision.

1. Please cite Rowan et al G3 Rapid and Inexpensive Whole-Genome Genotyping-by-Sequencing for Crossover Localization and Fine-Scale Genetic Mapping, which reports a useful HMM for crossover detection from short read data - this has been quite widely applied in the plant field.

*We have included this citation in the Hidden Markov Model section [line 372].*

2. It is worth noting that the number of crossovers per chromosome per meiosis is usually low (1-2) meaning that large numbers of cells are required to build up a deep genetic map.

*We have added a sentence which addresses this point in the section, which now reads: “In eukaryotes, the number of crossovers is low per meiosis, therefore large numbers of sequenced gametes are required to create a high-density genetic map.” This is in the section “The iMap” [lines 134-135].*

3.1 Please make it clear that this won't work very well if an individual is quite homozygous.

*We have clarified that heterozygosity is required for linkage map construction and have added a sentence to the last paragraph of the section “Phasing”, which now reads: “It is worth noting that iMap construction - like all genetic map construction - is most useful for genomic regions of heterozygosity” [lines 359-361].*

3.2 “Just out of curiosity how heterozygous is the 'typical' human?”

*A human on average has 3-4 million heterozygous SNPs, which is equivalent to around 1 per 1,000 bases (Table 1 from https://doi.org/10.1038/s41586-021-03205-y). This number will vary depending across populations but provides a rough indication of the typical resolution possible for crossover detection in humans.*

*Since humans are outbreeding, there is limited opportunity for being homozygous at a given locus. Nevertheless, it does happen and the rate at which it occurs is a function of many factors including geographic location (e.g. small island with a small population) and customs. Pemberton et al 10.1016/j.ajhg.2012.06.014 estimate that runs of homozygosity range from 100 Mb to 1 Gb (~3% - 30%) of the human genome, depending on geography, of the typical resolution possible for crossover detection. Large runs of homozygosity (such as in individuals with a high degree of inbreeding) will limit the resolution of crossover detection.*

4. The main focus is on crossovers - please also discuss gene conversions and the relative difficulty of detecting them due to short tract lengths. My feeling is that very low coverage data, which single cell data often is, is unsuitable for detection of conversions. I realise the gene conversions are not as relevant for SV detection, but they are also an interesting repair outcome from meiosis that would be interesting to study with single gamete approaches.

*We agree that gene conversion will be difficult to detect with single cell genomic data for two main reasons given: 1) the low level of coverage with these single-cell technologies, and 2) the requirement for appropriately spaced polymorphisms (e.g. 1 variant per 1,000 nucleotides in humans, on average, makes this challenging). If these limitations could be overcome in the future it will be interesting for meiosis research and could extend works that are traditionally limited to meiotic double-strand break and crossover hotspots, or dependency on high levels of sequencing coverage of a trio (i.e. mum, dad, offspring).*

*A comment addressing this limitation has been included in the section “Platforms for constructing iMaps – proof of principle and opportunities: Challenges and opportunities”, which now reads: “With future improvements to sequencing depth and resolution it may be possible to detect gene conversion events, which would be beneficial for crossover hotspot research.” [lines 306-308].*

5. Are the authors aware of any examples of SV heterozygosity causing effects on crossovers in human pedigree or other classical genetic maps?

*There are reports of SV heterozygosity and other forms of genomic diversity affecting crossover distributions in a number of species including mammals, worms and plants. We believe that this further makes the case for the utility of iMaps both for de novo linkage map construction, and for basic research questions about crossover distributions.*

*Examples include:*

*● Morgan, Andrew P., Daniel M. Gatti, Maya L. Najarian, Thomas M. Keane, Raymond J. Galante, Allan I. Pack, Richard Mott, Gary A. Churchill, and Fernando Pardo-Manuel de Villena. 2017. “Structural Variation Shapes the Landscape of Recombination in Mouse.” Genetics 206 (2): 603–19. https://academic.oup.com/genetics/article/206/2/603/6064292*

*They observed large scale CNV suppresses crossovers from occurring nearby from whole-genome sequencing of a Diversity Outbred (DO) mouse population*

*● Hammarlund, Marc, M. Wayne Davis, Hung Nguyen, Dustin Dayton, and Erik M. Jorgensen. 2005. “Heterozygous Insertions Alter Crossover Distribution but Allow Crossover Interference in Caenorhabditis Elegans.” Genetics 171 (3): 1047–56.*

*They observed crossover distributions changed as an effect of a large-scale heterozygous insertion using Caenorhabditis elegans*

*● Ziolkowski, Piotr A., Luke E. Berchowitz, Christophe Lambing, Nataliya E. Yelina, Xiaohui Zhao, Krystyna A. Kelly, Kyuha Choi, et al. 2015. “Juxtaposition of Heterozygous and Homozygous Regions Causes Reciprocal Crossover Remodelling via Interference during Arabidopsis Meiosis.” eLife 4 (March). https://doi.org/10.7554/eLife.03708.*

*They observed that runs of homozygosity, adjacent to runs of heterozygosity, influence crossover positioning. This was performed with the model plant Arabidopsis thaliana.*

*● Pemberton et al 2012 “Genomic Patterns of Homozygosity In Worldwide Human Populations” The American Journal of Human Genetics 91, 275-292.*

*Similar to Ziolkowski (above), they observed that runs of homozygosity influence, adjacent to runs of heterozygosity, influence crossover positioning. This study was with humans.*

*● Leflon, Martine, Laurie Grandont, Frédérique Eber, Virginie Huteau, Olivier Coriton, Liudmila Chelysheva, Eric Jenczewski, and Anne-Marie Chèvre. 2010. “Crossovers Get a Boost in Brassica Allotriploid and Allotetraploid Hybrids.” The Plant Cell 22 (7): 2253–64.*

*They observed that an extra copy of chromosomes resulted in a large increase in crossover numbers in Brassica hybrids.*

Reviewer #2: The manuscript, through reviewing existing work, decries the idea of using single gamete sequencing to facilitate genetic map construction, which can subsequently help achieve haplotype-resolved assemblies for individuals, providing the basis for finding potential links between genetic variations and health. The authors explain technologies/methods and questions relevant to genetic map construction. Below lists my major and minor comments.

Major:

1. To avoid any misunderstanding, all concepts described in the manuscript need to be accurately defined. For example, what does genome structure (given in title), genetic structure (line 307) and haplotype structure (line 482) stand for?

*We have changed “genetic structure” to “genomic structure” [line 333]. Haplotype is defined in the glossary.*

2. The relationship between genome structure, genetic map and genome assembly need to be clearly and sufficiently explained.

*We have added further details at the start of the second paragraph of “Introduction”, which reads: “High throughput DNA sequencing has made genome assembly more accessible, however fragmented DNA sequences still need to be assembled into highly contiguous chromosomal sequences. Genome assembly has historically required a dense genetic map to anchor and orient short DNA sequences onto larger chromosome-scale fragments.” [line 39-42]*

3. Independent of a genetic map, highly continuous genome assembly can be achieved using other technologies, such as chromosome conformation capture (https://www.nature.com/articles/s41587-020-0711-0) or strand-seq (https://www.nature.com/articles/s41587-020-0719-5). Indeed, conversely, such assemblies can be viewed as genetic maps with a higher resolution than those built up with limited markers. Therefore, it would be necessary for the authors to discuss more on the advantages (and/or disadvantages) of using a genetic map to assist genome assembly, compared with other methods, to make the statements from line 37-54 reasonable.

*We have added citations to the above two papers in the second paragraph under the Introduction section [line 58]. We agree that highly continuous genome assembly can be achieved using multiple technologies. We have clarified that we propose the use of iMaps as a complementary tool in genome assembly and detection of structural variation. This has been amended to read:*

*“The iMap could therefore be used as a complementary tool for genome completion and gap closing.” This is in the section “The iMap” [lines 142-143].*

*We also say in the section “Conclusion and future perspectives”:*

*“We believe that high-throughput sequencing of gametes offers a tool that can complement some of the limitations of other sequencing technologies.” [lines 462-464].*

4. As stated by the authors, there are variations in individual genetic maps, regarding crossover distributions (in gametes) and structural variations. It would be interesting to illustrate/propose the ideas of how such variations can be recognized and compared in details (although already stated briefly in line 372-378). For existing methods, comprehensively categorizing them (probably with a table) with advantages and disadvantages would be more helpful for readers.

*We have elaborated on how variations in individual maps could be detected with standard quantitative methods. The relevant passage now reads:*

*“Inter-individual differences in meiotic crossover landscapes can be revealed by crossover density plots that plot distributions of crossover locations along chromosomes for all individuals. Statistical tests for differences in distributions of the number of crossovers detected per gamete within individuals can also be applied. Such tests can be used to test the effects of specific factors that may influence variation in individual genetic maps. Bootstrapping and permutation testing approaches are useful where assumptions required for standard statistical estimators are not met, for example when comparing total genetic map lengths or evidence for crossover interference between experimental groups. In addition, individualized crossover interference can be analyzed and compared within individuals (Bell et al. 2020).” and has been added to the section “Comparing genetic maps” [lines 411-420].*

*In our opinion, there are not enough specific methods that have been proposed/applied for this purpose to warrant a table so we prefer to describe the statistical approaches as we have in the passage above.*

5. It is doubtful that DNA molecule size and read density can be used to filter out "doublets" in single-cell sequencing (line 259-261). For example, how to reconstruct the molecules? It is not clear. However, if these would be true, could the authors explain ideas in details? And, besides "doublets", would "halflets" where DNA from the same cell are ligated with two different barcodes challenge the related analysis?

*We have clarified how doublets can be identified and excluded in the section “Platforms for constructing iMaps – proof of principle and opportunities: Challenges and opportunities”, which reads:*

*“Doublets can be identified for single-cell methods by metrics like heterozygosity (which is not expected when sequencing single haploid cells) and read number (more reads for a barcode are observed than would be expected for a single cell). For linked-read methods, the constructed DNA molecule size, coupled with read number per constructed molecule, provides an indicator of doublet probability (for linked-reads sequencing when two different HMW molecules, from adjacent genomic regions, are tagged with the same barcode and are incorrectly constructed as one continuous DNA molecule).” [lines 284-290]*

*“Halflets” are less likely to happen due to the nature of the bead-based methods. However when the DNA material from one cell is encapsulated in two droplets and associated with two different barcodes, these “halflet cells” will be filtered out by standard quality control steps as their profile looks indistinguishable from “poor quality” true singlet cells that have no read coverage for many chromosomes and thus fail standard QC checks.*

6. It would be interesting to discuss how feasible it is to build iMap in species with complex genome features, such as high levels of repeats.

*We agree that it will be interesting to build iMaps in species with highly repetitive genomes. In the second paragraph under “iMap”, we discuss that iMaps can assist in de novo assembly of large genomes along with the whole tool kit, such as long reads and possibly Hi-C. We believe that we have captured the answer to this question in the second paragraph of the section called “The iMap” [lines 152-167]. It reads:*

*“As chromosome numbers and ploidy increase, so too does the difficulty of finding linked markers for construction of a genetic map. Gamete-derived iMaps offer a valuable component in the genome assembly toolkit. An important proof of concept with apricot used a droplet encapsulation technique to sequence haploid gametes (Campoy et al., 2020). Using 445 gametes, SNPs were phased using genetic linkage. Next, bulk long-read sequences were mapped to their matching haplotypes. A number of orthogonal approaches verified that an accurate de novo genome assembly had been generated. We believe that such an approach should assist consortia that sequence large, complex and economically important genomes such as the 17 Gb allohexaploid bread wheat with 42 chromosomes (International Wheat Genome Sequencing Consortium (IWGSC), 2014; International Wheat Genome Sequencing Consortium (IWGSC) et al., 2018; Ling et al., 2013, 2018; Paux et al., 2008). Finally the same apricot haploid short-read dataset also detected non-allelic meiotic crossovers at low frequencies, which opens up useful future fertility-related applications in individuals of all species. Short-read technologies alone cannot accurately detect and assemble the genome of species with high levels of duplications and therefore genome assembly requires an integrated approach.”*

Minor:

Line 103-105: please explain why crossovers are needed for characterizing inversions.

*We have added a sentence to explain why crossovers are needed to detect inversions with genetic approaches. This has been added to the section “Genomic structural variation” [lines 96-104] and reads:*

*“Structural variation (SV) is by definition a variation in the marker ordering of a genetic and physical map (Figure 1C). SVs are genomic alterations that can range from chromosome-scale alterations down to smaller inversions, duplications, insertions, deletions and translocations. Historically, chromosomal-scale SVs have been detected with cytogenetic approaches and can still only be visualized when multiple megabases in size. An orthogonal approach to detect SV, such as inversions, by measuring crossovers is possible by observing changes in marker linkage that can only be resolved through a reordering of markers. However, advances in high-throughput sequencing and computational methods are improving detection and breakpoint resolution.”*

Line 124-149: "single-cell sequencing" is "single-cell whole-genome DNA sequencing", correct? Would there be other possibilities (related to lines 279-281), and would there be additional challenges?

*iMap methods do not have to use single-cell whole-genome DNA sequencing. Other possibilities could be used such as scATAC-seq, single-cell targeted panel resequencing, and scRNA-seq is not a method that we consider viable (in-house unpublished data, and recent Science paper, Bhutani et al 2021, confirming the cytoplasmic sharing of scRNA transcripts between four sperm cells from the same meiosis).*

Line 128-131: it needs to be elaborated more on how to achieve genome completion and gap closing using iMap. What would happen in highly repetitive regions like centromeres where there is nearly no recombination?

*We agree that this requires clarification. The iMap approach has limitations, like all approaches, and we clarify that it could be a complementary tool to what exists already by adding a couple of sentence to the section “The iMap” [line 143-147]. The new material reads:*

*“The advantage of the iMap approach can be the capacity to assay tens of thousands of samples from one individual, and likely more as sequencing costs come down. Therefore genomic regions that have extremely low crossover rates, e.g. centromeric regions, will still be challenging, but by assaying many gametes researchers can increase the probability of finding the desired recombinants to assist with gap closing.”*

Line 254-256: Could the authors explain the general idea on how to reconstruct "linear order of genetic markers for an individual" with COs detected in pooled gametes?

*Because there are only a limited number of high molecular weight DNA molecules per droplet (10x Genomics suggest aiming for ~10 gDNA fragments per bead), this results in a very low probability of the same region - from multiple gametes - appearing twice in the same bead. Therefore for a given region, the probability of a region being “recombinant” or “parental” allows for calculation of crossover frequencies between markers, which in turn permits the linear ordering of the markers.*

Line 266-268: For polyploids, would there be ways to filter out doublets?

*Doublet detection in polyploids will be more challenging than for diploids, but doublets could be detected for polyploid species, as per established workflows for diploid species by observing unexpected read numbers for a given barcode compared to the majority of barcodes. The detection of heterozygosity - or more accurately, multiple alleles - would be less useful as a filtering step in polyploids than it would for diploid organisms, because a polyploid gamete is expected to have multiple syntenic alleles of most genes.*

Line 377-378: What could be the measures on tuning window size and marker density? It's not informative by just saying "need to be managed carefully".

*We agree that more detail is required. We have expanded this remark in the section “comparing genetics maps”. It now reads “Different window sizes may be tried to identify crossover events with higher or lower resolution. Larger window sizes will enable more stable identification of the haplotype but reduce the precision of inferred crossover locations. Higher marker density and deeper read coverage allow the use of smaller window sizes. Downsampling analysis can be helpful in making sure the marker densities are at the same scale across all samples (Bell et al. 2020).” [lines 407-411]*

**Second round of review**

**Reviewer 2**

The authors have cleared up most of my questions, while still three of them need more efforts to clarify.

Last major 4. According to the manuscript, there are two types of variations in genetic maps (lines 64-69).

The methods that the authors have added is for investigating variations in recombination (line 417-426).

The methods for the other type - structural variations - are still not well explained. If the authors are not going to categorize the methods (in this review), information from existing review papers can at least be discussed, e.g., https://genomebiology.biomedcentral.com/articles/10.1186/s13059-019-1828-7.

In addition, the statement "crossovers needed for their (i.e., INVs) characterization" (line 115) still needs more clarification. What does "characterization" refer to? If it is only about "detection" of INVs (which should be the case by reading following line 112-113), this statement is incorrect especially this is discussed in the context of long-read sequencings. A simple example (against the statement) could be, giving that two haplotypes show a 10 kb INV and we sequence a 20 kb read from each of them fully covering the inverted regions, by simply comparing the two reads based on alignments, we can easily detect the INV. So, why should a crossover be required for the INV detection?

Perhaps more logically, this requirement should be discussed after the method for INV detection at line 100-102?

Last major 5. If any, please cite references for the proposed ways of processing doublets (line 277-290).

In addition, for the response of "Because there are only a limited number of high molecular weight DNA molecules per droplet (10x Genomics suggest aiming for ~10 gDNA fragments per bead), this results in a very low probability of the same region - from multiple gametes - appearing twice in the same bead. Therefore for a given region, the probability of a region being "recombinant" or "parental" allows for calculation of crossover frequencies between markers, which in turn permits the linear ordering of the markers.",

I still do not find that "..., which in turn permits the linear ordering of the markers" is clear. I agree within the same molecule (from one bead), a crossover could be detected. But what are the markers between which we can calculate crossover frequency? And more importantly, how does this (i.e., crossover frequency) allow the ordering of hundreds to thousands of markers within different beads at genome-scale? It would be helpful for readers if the authors can give more related methods and discussions regarding these questions in the manuscript.

**Authors Response**

**Point-by-point responses to the reviewers’ comments:**

Last major 4. According to the manuscript, there are two types of variations in genetic maps (lines 64-69).

The methods that the authors have added is for investigating variations in recombination (line 417-426).

The methods for the other type - structural variations - are still not well explained. If the authors are not going to categorize the methods (in this review), information from existing review papers can at least be discussed, e.g., https://genomebiology.biomedcentral.com/articles/10.1186/s13059-019-1828-7.

*We thank the reviewer for highlighting the discussion of methods for investigating structural variants. We think that a full categorization of such methods is out of the scope for this review, so we have added this reference and thank the reviewer for pointing us to it.*

In addition, the statement "crossovers needed for their (i.e., INVs) characterization" (line 115) still needs more clarification. What does "characterization" refer to? If it is only about "detection" of INVs (which should be the case by reading following line 112-113), this statement is incorrect especially this is discussed in the context of long-read sequencings. A simple example (against the statement) could be, giving that two haplotypes show a 10 kb INV and we sequence a 20 kb read from each of them fully covering the inverted regions, by simply comparing the two reads based on alignments, we can easily detect the INV. So, why should a crossover be required for the INV detection?

Perhaps more logically, this requirement should be discussed after the method for INV detection at line 100-102?

*We acknowledge that “characterization” was a misleading term, and have reworded this as “detection” as suggested. We have also specified that our example is given in the context of SVs > 100 kb, which is the rough SV size where iMaps start to provide information that is out of reach for current average long-read length sequencing approaches, and reflected in the size limits of the SVs detected by the cited studies Huddleston et al 2017 and Chaisson et al 2019 where the upper limits of SV detection were around 30 and 100 kb respectively. We thank the reviewer for the suggestion to move this section to line ~100, but on balance we have decided to keep it where it is and hope that with the suggested improvements the passage is now clear for the reader.*

Last major 5. If any, please cite references for the proposed ways of processing doublets (line 277-290).

*Citations for these doublet detection methods have been added.*

In addition, for the response of "Because there are only a limited number of high molecular weight DNA molecules per droplet (10x Genomics suggest aiming for ~10 gDNA fragments per bead), this results in a very low probability of the same region - from multiple gametes - appearing twice in the same bead. Therefore for a given region, the probability of a region being "recombinant" or "parental" allows for calculation of crossover frequencies between markers, which in turn permits the linear ordering of the markers.",

I still do not find that "..., which in turn permits the linear ordering of the markers" is clear. I agree within the same molecule (from one bead), a crossover could be detected. But what are the markers between which we can calculate crossover frequency?

*In this context – as with single gamete sequencing discussed earlier in the review - the markers are simply single nucleotide polymorphisms. Polymorphic markers would be identified with established variant calling methods and informative markers (I.e. those that distinguish between the parental haplotypes) can be used to phase an outbred individual’s genotypes as shown in Bell et al (2020) - phasing is a given for F1 hybrids of inbred strains of model organisms like mouse. Crossovers can be detected by sequencing HMW DNA molecules and observing a switch from one parental haplotype to another, just as we propose for single-gamete sequencing. Thus, crossover frequencies can be calculated between pairs of markers and the linear ordering of markers (I.e. construction of a genetic map) can be done using existing, established approaches.*

*Proof of this concept is shown in the following two references:*

*• Sun H, Rowan BA, Flood PJ, Brandt R, Fuss J, Hancock AM, et al. Linked-read sequencing of gametes allows efficient genome-wide analysis of meiotic recombination. Nat Commun. 2019 Sep 20;10(1):4310.*

*• Dréau A, Venu V, Avdievich E, Gaspar L, Jones FC. Genome-wide recombination map construction from single individuals using linked-read sequencing. Nat Commun. 2019 Sep 20;10(1):4309.*

And more importantly, how does this (i.e., crossover frequency) allow the ordering of hundreds to thousands of markers within different beads at genome-scale? It would be helpful for readers if the authors can give more related methods and discussions regarding these questions in the manuscript.

*Measuring crossover frequency in live progeny e.g. F2 mice, or a droplet with a high-molecular weight molecule e.g. from an F1 mouse, is fundamentally the same in that for a heterozygous region, some progeny (or high-molecular weight molecules) will be “parental” and some will be “recombinant”. The fraction which are recombinant, can be used with a mapping function to calculate the centimorgan distance between markers. The principles that Sturtevant used to order more than two markers on the same chromosome, can be equally applied with high-molecular weight read recombination fractions. While the computational complexity is drastically larger when ordering hundreds of thousands of markers, the same principles apply.*
